# Supplementary material for: A near-tight lower bound on the density of forward sampling schemes
Source: Bioinformatics. 2024 Dec 12;41(1):btae736. doi: 10.1093/bioinformatics/btae736 (PMC11676336; doi:10.1093/bioinformatics/btae736)
Supplement: btae736_Supplementary_Data [file btae736_supplementary_data.pdf]

A. (s, w, k)-multi-local schemes

Given a set A, we define $\binom{A}{s}$ as the set of all subsets of A of cardinality s.

Definition 8 A (s, w, k)-*multi-local scheme* corresponds to a sampling function $f : \Sigma^{w+k-1} \to \binom{[w]}{s}$, where s is the sketch size, w is the window guarantee, k is the k-mer size, and $\sigma = |\Sigma|$ is the alphabet size.

A quick substitution reveals that (s, w, k)-multi-local schemes are equivalent to (w, k)-local schemes when $s = 1$. These schemes enable a stricter window guarantee by ensuring that at least s k-mers are selected for every window of w k-mers. However, it was recently shown that this generalization can also be used to outline a more relaxed window guarantee (Kille et al., 2023).

Consider the case where instead of desiring at least one sampled k-mer for every w k-mers, the requirement is to have s k-mers sampled for every sw k-mers. This latter goal can be accomplished by a (w, k)-local scheme, but as was shown in Kille et al. (2023), a much lower density can be obtained with an (s, sw, k)-multi-local scheme.

Here, we will show how the bounds in the main text can be extended to yield a lower bound for (s, w, k)-multi-local schemes.

A (s, w, ℓ)-multi-UHS is defined as a mapping $\alpha : \Sigma^\ell \to [s + 1]$ that assigns weights to vertices in B_ℓ such that any sequence of w adjacent ℓ-mers must have a combined weight of at least s. Again, when $s = 1$, this corresponds to the (w, ℓ)-UHS described in the main text. We now provide an extension to Proposition 4 which provides a lower bound on the size of a (s, w, ℓ)-multi-UHS.

Proposition 9. *For any (s, w, ℓ)-multi-UHS α,*

$$\sum_{W \in \Sigma^\ell} \alpha(W) \geq \sum_{p|\ell} M_\sigma(p) \cdot \left\lceil \frac{sp}{w} \right\rceil.$$

Proof We first show that for any simple cycle of size p in B_ℓ, the combined weight of the cycle must be at least $\left\lceil \frac{sp}{w} \right\rceil$. Let $W_0, \ldots, W_{p-1}$ be the vertices in a cycle of length p. Consider the walk of length w along the cycle starting at W_0. We have that $\sum_{i \in [w]} \alpha(W_i) \geq s$ where indices are taken modulo p. This same inequality holds for all p unique paths of length w along the cycle and summing over all of them yields $\sum_{i \in [p]} \alpha(W_i) \geq \frac{sp}{w}$. Finally, since $\sum_{i \in [p]} \alpha(W_i)$ must be an integer and each pure cycle in $\mathcal{C}_\ell$ of length p corresponds to an aperiodic necklace of length p, of which there are $M_\sigma(p)$, we arrive at the result:

$$\sum_{W \in \Sigma^\ell} \alpha(W) \geq \sum_{x \in \mathcal{C}_\ell} \left\lceil \frac{s \cdot |x|}{w} \right\rceil = \sum_{p|\ell} M_\sigma(p) \left\lceil \frac{sp}{w} \right\rceil. \qquad \square$$

The multi-local schemes also require that we generalize our definition of a charged context. As a multi-local scheme can select multiple new k-mers in a single window, we change our binary notion of a charged context to a mapping of weights to contexts, where a context W has weight $\alpha(W)$ if $\alpha(W)$ previously unsampled positions are selected in the final window of length $w + k - 1$ in W. Similar to local schemes, a multi-local scheme's necessary context is the current window of w k-mers as well as the previous $w - 1$ windows, leading to a context size of $2w + k - 2$.

Lemma 10. *If f is a (s, w, k)-multi-local scheme and $\alpha : \Sigma^{2w+k-2} \to [s + 1]$ is the corresponding mapping of weights to contexts, defined as $\alpha(W) = |A \setminus B|$, where A consists of all positions in the context selected by the final window and B consists of all positions in the context selected in all previous windows, i.e.*

$$A = \{j + (w - 1) \mid j \in f(W[w - 1, 2w + k - 2))\}$$

$$B = \left\{ \bigcup_{0 \leq i \leq w-2} \{j + i \mid j \in f(W[i, i + w + k - 1))\} \right\},$$

then α is a $(s, w, 2w + k - 2)$-multi-UHS.

Proof Our proof follows a similar structure to that of Lemma 5. Let us show that the total weight of any path of length w in B_{2w+k-2} is at least s. Let a sequence of w consecutive contexts of length $2w + k - 2$ be given as $(W_0, \ldots, W_{w-1})$. Take S to be the sequence of length $3w + k - 3$ such that $S[i, 2w + k - 2 + i) = W_i$. Then we have that f on the last $(w + k - 1)$-mer of W_{w-1} (which is $S[2w - 2, 3w + k - 3)$) selects s distinct indices $i_1, i_2, \ldots, i_s$ in S where for each $1 \leq p \leq s$ we have $i_p \geq 2w - 2$. Suppose n of these indices are selected by f on any previous $(w + k - 1)$-mer of S, indexed $i_{q_1}, \ldots, i_{q_n}$, while the other $s - n$ i_p are not (meaning $\alpha(W_{w-1}) = s - n$). Clearly, if $n = 0$, we are done. Therefore, assume instead $n > 0$.

Recall that for all $1 \leq j \leq n$, $i_{q_j} \geq 2w-2$. Since $0 \leq f(\cdot) \leq w-1$, we have that for each j, the first $(w+k-1)$-mer $S[m_j, m_j+w+k-1)$ in S such that f picks the index i_{q_j} satisfies $w - 1 \leq m_j \leq 2w - 2$. Then for each j, the choice of m_j yields that $W_{m_j - w+1}$ selects a new location i_{q_j} when f is applied to its last $(w + k - 1)$-mer. This means that $\sum_{l \in [w-1]} \alpha(W_l) \geq n$, since the sum accounts for every context of the form $W_{m_j - w+1}$, and hence every chosen index $i_{q_1}, \ldots, i_{q_n}$. As $\alpha(W_{w-1}) = s - n$, we have that the sum of the weights of the w contexts $W_0, \ldots, W_{w-1}$ is at least s. $\square$

Tying these results together using the same line of reasoning as we did in the main text for local schemes, we arrive at a lower bound for multi-local schemes:

Corollary 11. *If f is a (s, w, k)-multi-local scheme, then*

$$\mathrm{d}(f) \geq \frac{1}{\sigma^{2w+k-2}} \sum_{p|2w+k-2} M_\sigma(p) \left\lceil \frac{sp}{w} \right\rceil.$$

Let us consider the use case of multi-local schemes described earlier, i.e., where the requirement is to have s k-mers sampled from every sw k-mers. We showed in the main text that this can be accomplished by a (w, k)-forward scheme with density at least $g_\sigma(w, k)$, or a (w, k)-local scheme with density at least $g_\sigma(w, w + k - 2)$. With our new bound, we can see that the requirement can be achieved with a (s, sw, k)-multi-local scheme with density at least $g_\sigma(w, k + (2s - 1)w - 2)$. In other words, the lower bound for local schemes is the same pattern as the forward bound, but "shifted left" by $w - 2$, and the bound for multi-local is the same as the local bound, but again shifted left by $(2s - 2)w$.

B. An alternative form of $g_\sigma(w, k)$

When all divisors of ℓ apart from 1 have the same remainder modulo w, we can simplify $g_\sigma(w, k)$.

Corollary 12. *Let $N_\sigma(\ell)$ denote the number of cycles in the pure cycle partitioning of B_ℓ. Let w, ℓ be a pair of integers such that w does not divide ℓ and for all divisors $d \mid \ell$ excluding the unit divisor* 1, $d \equiv z \pmod w$). *Then for any (w,ℓ)-UHS U,* $|U| \geq \frac{\sigma^\ell + N_\sigma(\ell)(w-z)+\sigma(z-1)}{w}$.

Proof There are σ singleton cycles in a De Bruijn graph on an alphabet of σ characters, and each of these must be included in any hitting set U. For all remaining cycles, we have that $\lceil |c|/w \rceil = (|c| + w - z)/w$.

$$\begin{aligned}
|U| &\geq \sum_{c\in\mathcal{C}_\ell} \left\lceil \frac{|c|}{w} \right\rceil \\
&= \sigma + \left(\sum_{c\in\mathcal{C}_\ell} \frac{|c| + w - z}{w} \right) - \sigma \frac{1 + w - z}{w} \\
&= \sigma \left(1 - \frac{w - z + 1}{w} \right) + \sum_{c\in\mathcal{C}_\ell} \frac{|c|}{w} + \sum_{c\in\mathcal{C}_\ell} \frac{w-z}{w} \\
&= \sigma \left(\frac{z-1}{w} \right) + \frac{\sigma^\ell}{w} + N_\sigma(\ell)\frac{w-z}{w} \\
&= \frac{\sigma^\ell + N_\sigma(\ell)(w-z) + \sigma(z-1)}{w}. \qquad \square
\end{aligned}$$

While the formula in Corollary 12 still includes a summation over divisors due to its use of N_σ, it no longer involves any ceiling calculations. Furthermore, it shows that $g_\sigma(w,k)$ can be written as the following when w and k satisfy the constraints of Corollary 12

$$g_\sigma(w,k) = \frac{1}{w} + \frac{N_\sigma(w+k)(w-z) + \sigma(z-1)}{w\sigma^{w+k}}.$$

This form, compared to the general form of $g_\sigma(w,k)$, provides a more interpretable characterization of the gap between g_σ and $\frac{1}{w}$.

C. ILP Model

Forward ILP definition. We used Gurobi (Gurobi Optimization, LLC, 2024) to implement our ILP. While the basic model described below is sufficient, we made multiple improvements which enabled identifying solutions for larger σ, w and k. We use x_u to model $f(u)$, i.e. the index of the k-mer selected in the window $u \in \Sigma^{w+k-1}$. The $y_{u,v}$ variables represent the charge of a context of two adjacent windows u and v.

Given a De Bruijn graph $B_{w+k-1} = (V,E)$, the windows correspond to the vertices of the graph, and the contexts correspond to the edges. An edge (u,v) is not charged if $x_u = x_v + 1$. For a forward scheme, $x_u \leq x_v + 1$ for every edge (u,v). Therefore, we can define an ILP which minimizes the density of a (w,k)-forward scheme as follows:

$$\text{minimize} \sum_{(u,v)\in E} y_{(u,v)}$$

such that

$$\begin{aligned}
x_u &\in [w] && \forall u \in V, \\
y_{(u,v)} &\in \{0,1\} && \forall (u,v) \in E, \\
x_u &\leq x_v + 1 && \forall (u,v) \in E, \\
y_{(u,v)} = 0 &\implies x_u = x_v + 1 && \forall (u,v) \in E.
\end{aligned}$$

Local ILP definition. While we could define an ILP for local schemes similarly, the resulting model is inefficient due to the reliance on intermediate variables. Instead, we leverage the fact that the expected density on a random string (Marçais et al., 2017) is the same as the density of a (w,k)-local scheme on a circular De Bruijn sequence of order $2w+k-2$.

Let S be circular De Bruijn sequence of order $2w+k-2$, i.e. S is a circular sequence of length $L = \sigma^{2w+k-2}$ which contains every $(2w+k-2)$-mer exactly once. As S is circular, we note that when $i > j$, the substring $S[i..j)$ corresponds to $S[i..L)S[0..j)$, We use x_W to represent the value of $f(W)$ and y_i to be a binary variable which corresponds to whether or not position i was sampled. We define an ILP which minimizes the density of a (w,k)-local scheme as follows:

$$\text{minimize} \sum_{i\in[L]} y_i$$

such that

$$\begin{aligned}
x_W &\in [w] && \forall W \in \Sigma^{w+k-1}, \\
y_i &\in \{0,1\} && \forall i \in [L], \\
x_{S[i,(i+w+k-1) \bmod L)}{=}j &\implies y_{(i+j) \bmod L}{=}1 && \forall i{\in}[L],\ j{\in}[w].
\end{aligned}$$

Improvements to the base forward ILP. First, we added the additional constraint that each pure cycle c in B_{w+k} must have at least $\lceil |c|/w \rceil$ nodes which correspond to charged edges in B_{w+k-1}. This additional constraint helps substantially when $k \equiv 1 \pmod w$. In cases where $k \not\equiv 1 \pmod w$, we add corresponding constraints for a subset of simple cycles in B_{w+k} that correspond to pure cycles in higher or lower order De Bruijn graphs. While this process adds many more constraints to the model, it narrows the search space substantially and also increases the objective of the ILP linear relaxation, leading to a smaller (and sometimes nonexistent) integrality gap.

As adding this constraint requires the model to have variables representing the charge of a context, it was more efficient to directly use the context charges as the objective variable as opposed to using selected positions in a De Bruijn sequence (as we do in the local ILP). For the local ILP, the g_σ bound is relatively loose and therefore the aforementioned cycle constraints do not help much. Furthermore, modeling context charges requires more intermediate variables in the local ILP, hence we directly used sampled positions in a De Bruijn sequence as the objective for the local ILP.

If $g_\sigma(w,k)$ is tight for some σ, w, k, then the objective value of the linear relaxation of the ILP is the same as the integer objective value. As a result, the objective bound is fixed from the start. We leveraged this fact by telling the optimizer to focus on identifying integral solutions as opposed to decreasing the gap between the objective bound and objective value in cases where we suspect that $g_\sigma(w,k)$ is tight. This was done through the `heuristics` parameter.

Finally, we used previously computed solutions to provide starting points for the ILP optimization. Let f be a (w,k)-local scheme. We constructed a $(w+1,k)$-local scheme or a $(w,k+1)$-local scheme f' through ignoring the last character in an input window W of length $w+k$, i.e. $f'(W) = f(W[0,w+k))$. This is similar to the "naive extensions" described in Marçais et al. (2018). If we had a minimum density $(w-1,k)$-forward scheme or a $(w,k-1)$-forward scheme, we seeded the optimizer with the

"extended" sampling function which corresponded to whichever one of the precursors schemes yielded the lower density.

We ran our ILP model on a server with 128 threads and 128GB of RAM on all combinations of $2 \leq w \leq 12$, $1 \leq k \leq 12$, and $2 \leq \sigma \leq 4$. For each set of parameters, we limited the runtime to 12 hours. The ILP identified optimal forward schemes for 60 different sets of parameter and optimal local schemes for 11 different sets of parameters.

D. Additional figures

See Fig. 4 and Table 2.

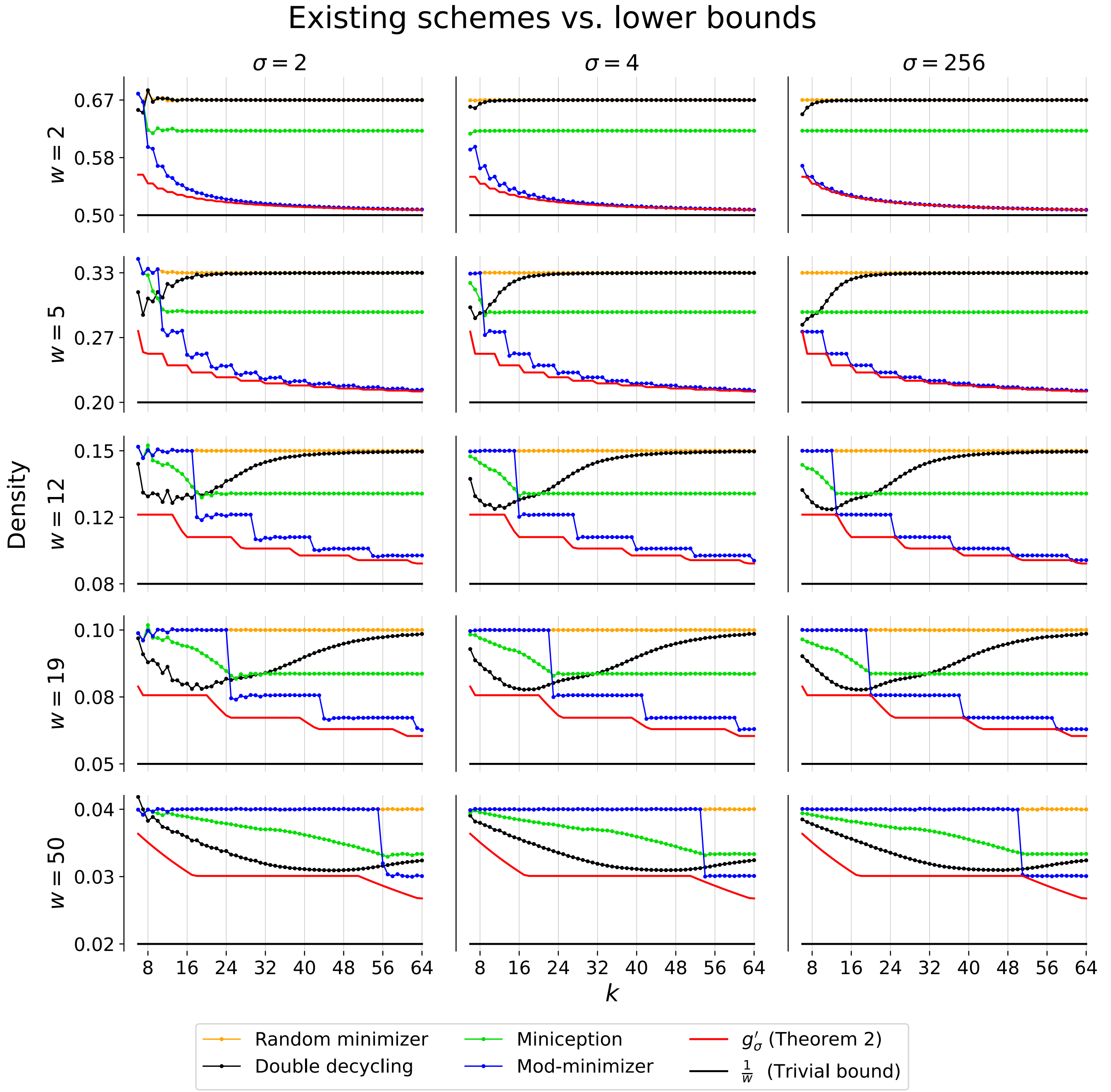

Fig. 4: Comparison of existing schemes to lower bounds. Densities were calculated by applying each scheme to a random sequence of 10 million characters and are plotted as solid dotted lines. The mod-minimizer uses parameter $r = 6$ for $\sigma = 2$, $r = 4$ for $\sigma = 4$, and $r = 1$ for $\sigma = 256$. Miniception uses parameter $\max(r, k - w)$. Lower bounds are plotted as solid lines.

Table 2. Our $g'_\sigma(w,k)$ lower bound on the density of forward schemes, and minimum densities of forward and local obtained by the ILPs described in Supplement C. Entries with an equals sign $(=)$ correspond to parameters where the minimum density is equal to the preceding column. Entries with the less-than-or-equal-to sign $(\leq)$ correspond to parameters where the local ILP identified a solution with lower density than the optimal forward scheme but timed out before determining whether the identified solution was optimal. Bold entries indicate cases where the local scheme is better than the $g'(w,k)$ bound for forward schemes. For $w=2$, all local schemes are forward by definition and therefore the minimum $(2,k)$-local scheme density is equal to the minimum $(2,k)$-forward scheme density. Empty cells correspond to parameters where the local ILP timed out before it was able identify a local scheme with lower density than the optimal forward scheme or prove that no such scheme exists. In these cases, it is unknown whether or not there exists a local scheme with lower density than the optimal forward scheme.

(a) $\sigma = 2$

w,k	$g'_\sigma(w,k)$ Fwd. bound		Minimum d(f) Forward		Minimum d(f) Local	
2, 1	0.750	3/4	=		=	
2, 2	0.625	5/8	=		=	
2, 3	0.625	5/8	=		=	
2, 4	0.578	37/64	=		=	
2, 5	0.578	37/64	=		=	
2, 6	0.559	143/256	=		=	
2, 7	0.559	143/256	=		=	
2, 8	0.546	559/1024	=		=	
2, 9	0.546	559/1024	=		=	
2, 10	0.539	1103/2048	=		=	
2, 11	0.539	1103/2048	=		=	
3, 1	0.562	9/16	=		=	
3, 2	0.438	7/16	0.469	15/32	=	
3, 3	0.438	7/16	0.453	29/64	=	
3, 4	0.438	7/16	=		=	
3, 5	0.401	411/1024	0.406	13/32	=	
3, 6	0.401	411/1024	0.402	103/256		
3, 7	0.401	411/1024	=			
3, 8	0.385	197/512	0.386	395/1024		
3, 9	0.385	197/512	0.385	1577/4096		
3, 10	0.385	197/512	=			
4, 1	0.438	7/16	=		=	
4, 2	0.359	23/64	0.375	3/8	0.371	95/256
4, 3	0.336	43/128	0.352	45/128	=	
4, 4	0.336	43/128	0.340	87/256		
4, 5	0.336	43/128	=		$\leq$ **0.335**	**687/2048**
4, 9	0.308	1261/4096	=		$\leq$ **0.308**	**10087/32768**
5, 1	0.359	23/64	=		**0.355**	**91/256**
5, 2	0.297	19/64	0.305	39/128	=	
5, 6	0.273	35/128	=		$\leq$ **0.273**	**2239/8192**
6, 1	0.297	19/64	=			
6, 2	0.258	33/128	0.262	67/256	$\leq$ 0.261	267/1024
7, 1	0.258	33/128	=		$\leq$ **0.257**	**263/1024**
8, 1	0.227	29/128	=			
9, 1	0.202	207/1024	=			
10, 1	0.183	187/1024	=			
11, 1	0.168	687/4096	=			
12, 1	0.154	631/4096	=			

(b) $\sigma = 3$

w,k	$g'_\sigma(w,k)$ Fwd. bound		Minimum d(f) Forward		Minimum d(f) Local	
2, 1	0.704	19/27	=		=	
2, 2	0.605	49/81	0.630	17/27	=	
2, 3	0.605	49/81	=		=	
2, 4	0.572	139/243	0.578	421/729	=	
2, 5	0.572	139/243	=		=	
2, 7	0.556	10939/19683	=		=	
3, 1	0.519	14/27	=		=	
3, 2	0.429	313/729	0.457	37/81		
3, 4	0.429	313/729	=		$\leq$ **0.428**	**2810/6561**
4, 1	0.407	11/27	=			
5, 1	0.337	82/243	=			
6, 1	0.287	209/729	=			
7, 1	0.251	548/2187	=			
8, 1	0.222	4379/19683	=			

(c) $\sigma = 4$

w,k	$g'_\sigma(w,k)$ Fwd. bound		Minimum d(f) Forward		Minimum d(f) Local
2, 1	0.688	11/16	=		=
2, 2	0.602	77/128	0.621	159/256	=
2, 3	0.602	77/128	=		=
2, 5	0.572	2341/4096	=		=
3, 1	0.508	65/128	=		
4, 1	0.402	103/256	=		
5, 1	0.334	685/2048	=		
6, 1	0.286	1171/4096	=		
